# Tukdam, Different Ontological Bodies, and Making Tibetan Deaths Visible

**DOI:** 10.1007/s11013-024-09889-x

**Published:** 2025-07-16

**Authors:** Donagh Coleman

**Affiliations:** University of California Berkeley, Berkeley, USA

**Keywords:** Ontology, Incommensurability, Cross-cultural philosophy, *Tukdam*, Documentary film

## Abstract

Unknown to science until recently, the postmortem state of *tukdam* has not yet been adequately explained in biomedical terms. One way to understand this phenomenon is through ideas of different cultural bodies and death processes, where *tukdam* emerges as a particular kind of Indo-Tibetan death. This article draws upon medical anthropological scholarship and literature in history of science and cross-cultural medicine looking at epistemological theories of perception where different ways of conceiving-perceiving and attending to the body contribute toward producing different medical bodies—and deaths. This epistemological approach has entailed the idea of one reality “out there,” which I call into question. I argue instead for an ontological approach, where epistemology and ontology are collapsed so that different forms of conceiving-perceiving contribute toward different forms of being. Such an approach seems apt in the case of different cultural bodies and death processes that we encounter with *tukdam* and other extraordinary Tibetan death displays. I explore Yogācāra Buddhist philosophy and its Tibetan appropriations along with science studies, medical and ontological anthropology to sketch out theory for how ontologically distinct bodies might come about. Tantric Buddhist bodies and deaths emerge as largely incommensurable with, and invisible to, the modern medical gaze with its attendant Euroamerican regime of truth where visibility, quantification, and technological measurability set the grounds for the real. This regime also dominates in the European documentary film world, as I discovered while making a documentary on *tukdam.*

## Introduction

In *tukdam* (Tib., *thugs dam*), advanced Tibetan Buddhist meditators die in meditation. Their bodies do not show the usual signs of death for days or even weeks after their clinical deaths. They appear lifelike, and some even remain sitting upright in meditation posture. Warmth is frequently reported around the heart area. According to Tibetan Buddhism, the meditators are resting in a subtle state of consciousness with an associated subtle material energy present in the body, and so are still in the process of dying. Yet according to modern biomedical and legal definitions, they are dead.^1^ As a biological and cultural phenomenon, *tukdam* disrupts normative Western categories of life and death, mind and body, and offers a fascinating focal point through which to explore such delineations and the incommensurability of what appear to be culturally scripted biologies.^2^

*Tukdam* is a relatively new phenomenon to science not yet adequately explained in biomedical terms. It makes sense within a Tibetan Buddhist framework however, where detailed explanations, signs, and so forth are given. In addition to delving into biomedical definitions, institutional practices and problematics around death, brain death, personhood and consciousness, on the science side I have followed the Tukdam Study led by the Center for Healthy Minds (CHM) at the University of Wisconsin-Madison. I also video-documented parts of the Study, which features as a strand in the documentary film *Tukdam: Between Worlds* that I developed, directed, and co-produced alongside my academic research.^3^ In my research and filmmaking, I have been stuck by the incommensurability of Tibetan and conventional scientific approaches, with their different background assumptions, epistemologies, and even ontologies regarding the body and mind, life and death. This article, then, is oriented from a position of incommensurability*,* and how we might think through this. Instead of joining the laudable effort of building bridges, I try to come to grips with radical difference.

My contemplation on incommensurability is also a response to presentations on the scientific *tukdam* research in our 2021 American Anthropological Association panel^4^ that formed the basis for this Special CMP Collection, as well as in an online mini-conference on death in Tibetan traditions organized by Yale University later the same year.^5^ In these events—and her contribution to this Special Collection—Tawni Tidwell uses the metaphor of “landscape” for the object of study, in this case *tukdam,* that scientific research and Tibetan traditions interpret and represent in different ways through their differing tools and methods of investigation (thereby producing different “maps” of the same “identical” terrain). As Tidwell frames it,

The Tukdam Study has been like a psychophysiological expedition where two field teams traverse identical landscapes with one team [neuroscientists, forensic pathologists and other scientists] in a thick-walled vehicle with the highest technology to measure altitude, slope, inclination, moisture, and so forth to navigate. Whereas the other team [Tibetan Buddhist adepts and physicians] is out in the elements fully exposed but able to directly see every microscopic change in vegetation, geography, weather, and incident… The two traditions can speak to one another about their landscape, but they feel as if these landscapes are not identical because both exploratory means or investigative vehicles with which they journey across the landscape are distinct and the maps each have augmented also now quite different (2023; this Special Collection).

I take Tidwell’s characterization (of the same landscape interpreted or perceived in different, perhaps complementary ways) to epitomize an epistemological approach. Resonating with decades of formidable scholarship on different medical systems and embodied cultural worlds (some of which I touch on below), such an approach is persuasive and generative, especially when put to the service of building bridges between different cultural knowledge systems, as Tidwell does. While acknowledging the power of such epistemological thinking, I bring attention to its limitations and argue for an ontological anthropological approach that trades more in alterity, as expressed in different cultural ontologies or worlds. As I hope to show, such an approach also seems apt in the case of different cultural bodies and death processes that we encounter with *tukdam* and other extraordinary Tibetan death displays.

The phenomenon of *tukdam* and conversations around it serve as departure points for my article, which is less concerned with *tukdam* or the Tukdam Study per se than it is with theorizing different ontological bodies and death processes, how these might be conceived to come about, and implications all this might have for commensurability. Although my focus is theoretical as opposed to ethnographic,^6^ such a short article cannot make an exhaustive argument for ontological anthropology or defend its many shades and strands. It could instead be considered as an intellectually productive *provocation* in the tradition of Eduardo Viveiros de Castro or Martin Holbraad. These ontological anthropologists also call for the decolonization of thought, which means taking the ideas, accounts, and entities of other cultural worlds seriously in their own terms *in a fundamental, ontological sense*, without being compelled to explain or validate them in terms of our Euroamerican natural or social sciences. It can also mean utilizing concepts and theories from outside our familiar Western repertoire in creative ways, in a play of “speculative metaphysics,” to put it in terms of Viveiros de Castro, who utilized Amerindian concepts to rethink philosophy and anthropological theory (2014). As our subject emanates from a Tibetan Buddhist world, it seems reasonable also to employ theory from this world in our project. In doing so, we will see another good reason for employing ontological anthropology, as hitherto unexplored resonances between ontological anthropological theory and strands of Buddhist philosophy come to light. Putting recent ontological scholarship into productive dialogue with Buddhist philosophy constitutes a novel contribution in philosophical anthropology. Mindful of the proliferating and often politicized uses to which “decolonizing” is put nowadays, here I claim its use mainly in this abstract, rather rarefied sense: using Buddhist philosophical theory as theory.

Theoretical considerations concerning different cultural worlds, bodies, and death processes lay the ground for observations on how different ontological bodies and deaths are rendered visible or invisible in different regimes of truth or evidence. Important (Tibetan) aspects of *tukdam* may be invisible to modern science with its attendant Euroamerican ontology, as well as to modern publics and state authorities where such an ontology dominates. Interactions with media gatekeepers involved in the production of my *tukdam* documentary illustrate challenges of making non-biomedical, in this case Indo-Tibetan, bodies and deaths visible.

## Tukdam: Between Worlds

Let me begin with a few scenes from *Tukdam: Between Worlds*^7^*,* my 2022 documentary film that juxtaposes Tibetan accounts of *tukdam* and the Center for Healthy Minds scientific study. As also implied by the film title, we are led into largely incommensurable worlds, inviting attempts to bridge or translate between the obvious differences in epistemologies and ontologies encountered. To illustrate the dialogue, if it can be called such, between the worlds, I will describe a sequence of scenes beginning a little after the documentary’s midpoint. We start with an interview with Dylan Lott (anthropologist and manager of the Tukdam Study at the time of filming in 2019), and his account of how the scientists were looking to explain the extraordinary outward signs of *tukdam*:

When we collect EEG data, we do two auditory protocols to ascertain whether or not there’s some activity… The contention is that at least for a time after death, there’s some residual brain activity in the brain stem that’s responsible for the integration of the body. In the absence of that, then we have a different question, another challenging question: If there is no low-level residual activity in the brainstem in those we’re observing in the state of *tukdam*, then how do we explain this delay in decomposition?

Cut to an interview with Tibetan Medicine doctor Ngawang Jinpa, a Men-Tsee-Khang (Tibetan Medical and Astro-Science Institute) Tukdam Study collaborator, who is helping the scientists to collect data in Tibetan exile communities in India:

I am not satisfied with the current research methods of modern science. Why not? The current method of modern science is to attach cables to the head and analyze brain waves. They analyze brain waves, but we are not claiming anything about brain waves. They have ceased. Buddhism and specifically the tantric tradition agrees that death has already occurred. Therefore I don’t think that the brain has much influence on *tukdam*.

Another thing is they take readings under the arms. They take the skin temperature. I’m not so sure about this. What we are focused on is the heart. Whether there is a presence of warmth at the heart and whether the subtle mind is still active in the heart or not.^8^ How could they determine anything about a state of *tukdam* through skin temperature?

After a visual interlude with images of Tibetan medical thangkas (paintings) illustrating a tantric Indo-Tibetan body with its subtle channel system also implicated in the death process and *tukdam,* the dialogue is continued by David Perlman. A neuroscientist and engineer who designed the initial portable measurement kit, Perlman was the Tukdam Study manager before Dylan.^9^

So there is the warmth around the heart. And there were four cases during the time that I was involved. And we only got clear thermal imaging data one of the times. And they were stone cold. They, you know, you could see very clearly in the thermal imaging that there is the wall [behind the body] and then the body. And everything is all the same color. There was no variation whatsoever. And every day, a group of devotees would come in and would carefully inspect the person and they would feel carefully with the back of their hand [around the heart area]. And they would be commenting on it, and then they would agree that the person was still in *tukdam*.

…When the Tibetan tradition talks about *tukdam*, they don’t have thermal imaging cameras. So when they say the body stays warm, the specific statement that the tradition is making is that when you touch the body, around the heart, you experience the feeling of warmth. Everything that I’ve ever experienced is completely consistent with the idea that people experience *tukdam* exactly the way the tradition says that people experience *tukdam*. …Just the more time that I spent on this project, the more striking was the contrast between, as long as you weren’t trying to do a whole bunch of hard science with the equipment, everything looked one way. And then as soon as you start bringing the equipment in and trying to nail everything down and take a bunch of physical measurements, then it all falls apart.

Tibetan medicine doctor Ngawang Jinpa highlights incommensurability, as does David Perlman, a “hard” scientist by training. Also implied in this cinematic exchange are different approaches to knowledge production: its technological mediation (through thermal cameras, EEG, etc.) in the case of modern science, whereas Tibetan knowledge is more tied to embodied evidence of finely tuned senses, or contemplative experience developed in part through different kinds of internalized ritual enactments, and scriptural sources.

In what follows, I will consider in some detail how deep this incommensurability may run, and to what extent we might consider Tibetan bodies and death processes as different ontological realities—as opposed to different epistemological representations of the same “landscape” out there. What I mean by “ontology” and “epistemology” is influenced by (ontological) anthropological and science studies uses of these concepts, as will be further clarified and refined in below sections. To start with, ontology can be understood in terms of what *is* for us as *anthropos*—although this could be extended to other beings, or to multispecies worlds—in terms of phenomenal experience. Moving into more philosophical territory, we will see that it may be difficult to reach an ontological ground more fundamental than this. The distinction between epistemologies and ontologies might seem like terminological sophistry, yet what is at issue is a profound philosophical difference relatable to views of naturalistic realism on the one hand, and a kind of multinaturalism—or even irrealism or idealism, as some Buddhist philosophers would have it—on the other. This has implications for what it means to take different cultural worlds—and deaths— “seriously on their own terms” (which has, of course, been a core concern of any ethical and attentive anthropology for quite some time) and possibilities of their commensuration or translation. The following theoretical sections then constitute the main body and contribution of this text.

## Different Cultural Bodies and Deaths

Medical anthropology offers productive ways to approach the problem of incommensurability. In simple terms, different ways of attending to the body and its death process produce different bodies and deaths. Vincanne Adams, for instance, has shown how internally consistent and efficacious Tibetan medical theories and clinical practices have been, conceived as they are in ways radically different from biomedical understandings,^10^ and has applied Margaret Lock’s concept of local biologies (where cultural and social norms produce different biology) to Tibetan bodies.^11^ In Adams’ treatment, local biology can be understood on several different levels. One of these relates to epistemological theories of perception—how different criteria for knowledge and cultural ways of conceiving-perceiving, as outlined in more detail below, produce different bodies and their processes. Taking this line of thought further, local bodies might even be considered as distinct ontological realities.^12^

Such a radical claim is associated with so-called “ontological turn” thinking, where a modern Western nature/culture divide is abandoned along with notions of a culture-free nature “out there” on which we inscribe interpretations. While this school of thought is rather disparate with different lineages and articulations,^13^ it is often associated with science and technology studies and the work of anthropologists like Annemarie Mol, Bruno Latour, John Law, Martin Holbraad, Morten Pedersen, and Casper Bruun Jensen, as well as with modern ethnography and anthropological theorizing that emerged from work in the Amazon (e.g., Philippe Descola, 2013; Eduardo Viveiros de Castro, 2014). Ontological anthropologists argue that prior to their interventions, much of anthropology has been conducted as an epistemological exercise. Following a dominant Western tendency (or “ontology” in Descola’s terms^14^), reality has been assumed to be one, which is then known, represented, interpreted, or enacted in different ways by different social groups or cultures. Within the sphere of modern Euroamerican ontology, it is generally held that this “nature” is most accurately described by our natural science, while the human and social sciences come to grips with “culture.” Anthropology, as traditionally conceived, has then investigated differing cultural representations or systems, or epistemologies.^15^ In the ontological turn, anthropological theory shifts “from questions of knowledge and epistemology towards those of ontology,” as Henare, Holbraad, and Wastell declare in what is generally regarded as one of the foundational ontological turn texts, their introduction to *Thinking Through Things* (2007, p. 8).

What makes the ontological turn quintessentially anthropological, according to thinkers like Holbraad and Viveiros de Castro, is that here ethnographic data determine *ontologies* (in the plural). Instead of interpreting peoples and practices encountered in the field according to a modern Western naturalist ontology or academic theories, we take their worlds seriously on their own terms in a fundamental, ontological sense. This has the consequence of multiplying ontologies, which can be roughly equated with cultural worlds. In Holbraad and Pedersen’s formulation, “The ontological turn is not so much a matter of ‘seeing differently,’ in other words. It is above all a matter of seeing *different things*.”^16^ Such an approach calls into question a unitary nature (again, which we might assume science to be best approximating); the ontologists instead propose a multiplicity of realities calling to mind Viveiros de Castro’s Amerindian-derived concept of “multinaturalism.”^17^

As an example of ontological thought pertinent to our problem of incommensurability, we could turn to Annemarie Mol’s much-celebrated book *The Body Multiple: The Ontology of Medical Practice.* Here Mol shows how the perception of the body, and in particular the seemingly single medical condition of atherosclerosis of the leg arteries differs widely depending on the practices and training of the specialist and the technologies used to observe the phenomenon. For vascular surgeons, atherosclerosis comes into being through patients in their clinics making certain complaints (such as feeling pain while walking) and the surgeons finding a weak pulse in the leg. Pathologists, in contrast, enact atherosclerosis by preparing slices of amputated legs with dyes and looking at these under microscopes to find evidence of blocked leg arteries. Radiologists, hematologists, or epidemiologists enact atherosclerosis in yet other ways. Surprisingly, it is not that the different specialists are looking at different aspects of one thing, like blind men tracing out different parts of an elephant and coming up with widely divergent descriptions. Mol shows how their ways of seeing and describing fail to add up to anything coherent. In what I take to be a signature ontological move, the constitution of atherosclerosis—or bodies—is not a question of epistemology, of perceiving or representing a shared, objective reality, nature or landscape “out there” in different ways. In Mol’s praxiographic approach, practices enact objects that do not exist prior to them.

There is no single atherosclerosis of the leg arteries out there; it does not exist as a thing independent of the practices through which it is enacted, along with an associated assemblage of technologies and objects such as slides, dies, knives, microscopes, x-ray machines, patient files, questionnaires feeding into statistics, and so forth. None of the objects or actors—such as the surgeon—involved in atherosclerosis are single, solid characters either. After more investigation, they all appear to be multiple. In this way, Mol ends up with a rather good approximation of Buddhist dependent origination (not that she calls it such), and its flipside, *śūnyatā* or emptiness (lack of any inherent, independent, substantial existence). Here, the very existence of entities depends in part on our conceptual designation and delineation of them. Drawing on a Tibetan Buddhist Geluk interpretation (in a Mādhyamaka philosophical context), dependent origination (or arising) can be understood on three levels: the arising of things through causes and conditions, things as wholes arising dependent on parts, and finally their delineation or existence as dependent on conceptual designation, mental labeling, or imputation.^18^

In the context of this article’s cross-cultural philosophical ambitions, it should not escape our attention that much scholarship associated with ontological thinking in science and technology studies (STS) and medical anthropology (beyond Mol) articulates something similar to Buddhist dependent origination, which can also be seen to refuse distinctions of nature and culture.^19^ Latour’s actor-network-theory is a case in point (e.g., Latour, 1987, 1988, 2005). Consisting of human and non-human actors, and encompassing pretty much everything including technologies, concepts, things like transportation systems and flows, power grids, and on and on, anything can be described as a network. Ontologically speaking, there is no absolute network; they depend on the point of view, or goal of what one is looking for, as do where their boundaries are drawn. Things can be seen as nodes in the network, or as networks themselves. In Buddhist terms, since no independent, substantial entities can be found from their own side, they are conceptually designated, or, we could say, delineated. Things are constituted by ever shifting relations of interdependence; it is we who carve out phenomena (according to our habitual tendencies and conceptual conventions) from these flat ontologies where none exist in their own terms.^20^ As another point of resonance, we could mention Stacey Langwick’s work on postcolonial healing practices in Tanzania (2011). Combining Byron Good’s notion of “objectification” with Mol’s ideas of enactment and bodies as events or moments of ontological coordination, Langwick shows how Tanzanian bodies, maladies, and treatments—both biomedical and “traditional”—consolidate particular assemblages of actors, moments in time, and structures of relation.

If ways of seeing seem to produce different realities even within the same broad framework (of biomedicine) as Mol shows, this is obviously so in the case of different cultural worlds and their systems of knowledge. We see this in Langwick’s work attending to generative processes of objectification of bodies and maladies in Tanzania, and in other scholarship on cross-cultural medicine. Historian of science Shigehisa Kuriyama’s book *Expressiveness of the Body* and its shorter published offshoots^21^ present a compelling case of how different patterns of seeing and practice can give rise to different concrete realities or empirical bodies by tracing out different, incommensurable Chinese and Greek Galleanic medical bodies. We are faced with the apparent paradox where two people can be looking at the same body yet see very different things. Kuriyama argues that the different ways that ancient Greeks and Chinese understood and perceived the body derived from different ways of conceiving and experiencing themselves from within, i.e., different concepts of self. While dealing with different bodies, both sciences can nonetheless be empirically grounded and medically efficacious. More recently, in their philosophical defense of a kind of scientific realism, Hubert Dreyfus and Charles Taylor highlight acupuncture as an empirical system that clearly “works,” although it has not been adequately explained in biomedical terms^22^—thereby challenging a scientific universalism, or unity of a natural scientific “nature.”

To continue with examples related to Chinese medicine, medical anthropologist Judith Farquhar looks at Chinese medical treatment for women’s infertility in contemporary China in her article *Objects, Processes, and Female Infertility in Chinese Medicine*. Disease is a process that unfolds in time and cannot be pinpointed to single anatomical features. Obstructed fallopian tubes, for example, are not accorded great significance in treating infertility, which is rather seen as a result of the complex interaction of various Chinese medicine body systems’ processes *unfolding in time*. Farquhar points out that Chinese medicine gets murky (from a Western point of view) only when “one attempts to fill its intellectual objects (patterns of pathological function) with [Western-based] anatomical content.”^23^ Here, we have a problem of translation, arising from a misguided attempt “to translate the diachronic into the synchronic and processes into structures.”^24^

According to Farquhar,

The persistent analysis of material existence in terms of visible structural features of discrete things has been both a triumph and a limitation of the Western medical worldview.^25^

Both Kuriyama and Farquhar show how Western medicine’s focus on visually apprehensible anatomical structures contributes toward a certain Western medical body.^26^ On the other hand, processes unfolding in time and via the patient’s own narrative, attending to touch or sensation, and different sensory modalities contribute toward different empirical bodies.

The cultural delineation of “biological facts” becomes especially apparent in problems of defining death. As Sharon Kaufman notes*,* “Death is known, defined and disputed through various forms of human activity that surround it. Thus death is not a natural phenomenon. It is made. It varies culturally and historically.”^27^ Within the sphere of biomedicine alone, even slight examination of problematics around death reveal how conventional, and blurry, it is. Its conventional nature is dramatically brought to the fore with the “death-in-life” existences of the brain dead, permanently comatose, or those in persistent vegetative state. According to Kaufman, patients in life-support maintained liminal states are “a cultural experiment in ways of knowing and defining what a person is.”^28^ It is partly due to there not being any medical consensus on what constitutes personhood, consciousness, subjectivity, death, or even criteria for persistent vegetative state, that such forms of “death-in-life” exist and are maintained in our hospitals. (A host of other socio-economic-cultural factors also feeds into this situation, as Kaufman examines.)^29^

Of course, this is not to say there are no legal or medical definitions of death in Western post-industrialized countries, where death is still commonly declared on the basis of the heart stopping and the cascade of events following from this. Scholars like Margaret Lock have shown how more recent definitions of brain death^30^ arose in part as a response to organ transplant technologies and demand. As such, the new definition of brain death is far from “natural,”^31^ especially as consciousness—also deemed culturally significant as determining personhood and death—still eludes neuroscience. Its nature and instantiation in the brain, and even the physical world in general, are hotly debated within science, as well as in Western philosophy of mind and science.

To conclude this section, there is nothing particularly new about attending to multiple ways of knowing and how these contribute toward different cultural bodies or deaths. Such epistemological observations have been made since early medical anthropological discussions, for instance, on medical pluralism. This approach has been crucial for appreciating and learning from difference, but in my reading seems to depend on a kind of non-cultural “ur-ground” or basis, which is difficult to establish (in part because all knowledge is mediated).

Building on epistemological thinking, I advocate collapsing epistemology and ontology so that different ways of conceiving-perceiving contribute toward different forms of being. This is quite different to the position of Kuriyama and medical anthropologists like Farquhar who are not contesting some basic shared materiality of the body “out there” but rather how it is perceived and acted upon. I will further develop the provocation that there is no landscape out there. In following sections, ontological turn and science studies theory will conspire with and give way to Buddhist philosophy to land us in such groundless ground.

## Indo-Tibetan Ontologies of Death

To bring the discussion back to the Tibetan Buddhist world, tantric Buddhist bodies that undergo *tukdam* deaths derive in part from attending to the body and mind intensively in particular directed ways, informed by contemplative traditions and techniques through which one comes to encounter certain affordances or empirical patterns.^32^ Indeed, it is important to note that we are not just dealing with different abstract conceptualizations or theories or discourses, but embodied, sensory, affective knowledge acquired through attending to the body and mind in particular ways that are not technologically mediated (in the manner that biomedical death for instance often is). In this context, *tukdam* emerges as a particular kind of Indo-Tibetan death.

All of this again calls into question notions of objectivity and the universal body, which anthropological and history of science literature argue to be relatively recent European ideas anyway.^33^ In terms of ontological anthropologists like Philippe Descola, what is being called into question is the nature-culture distinction. This is dramatically disrupted by *tukdam;* in it, culture is inscribed into nature or biology. Again, this is not a case of inscribing different interpretations or cultural scripts onto a reality best described by our natural and social sciences, which would be falling back on a modern European ontology (that has gone global). In line with Viveiros de Castro’s call for the “ontological self-determination of the world’s peoples,”^34^ an ontological anthropological approach allows us to take Tibetan deaths and bodies as fundamentally real as a biomedical account of death.

In *tukdam,* tantric Buddhist conceptions and practices around death seem to produce, or be reflected in, concrete, physical manifestations observable also to those from outside this particular cultural world. What we might call symbolic religious forms then seem to shape biological, even ontological realities. There are more extreme Tibetan Buddhist death displays where this is even more apparent. Some practitioners’ bodies are reported to shrink, so that a large man’s corpse is reduced to the size of a small eight-year-old boy within a week or so after dying. While rare, this is very much part of the tradition. I collected eye-witness accounts of this phenomenon from high lamas such as Sakya Trizin and Tai Situ Rinpoche, whose integrity I have no reason to doubt. In their religious world, to lie or deceive about such matters would constitute a serious “spiritual lie” with grievous karmic consequences. The dramatic shrinking of the physical body after death fits into Tibetan Buddhist theory, where it is related to the dissolution and purification of the elements. As other examples, we could mention different kinds of relics sometimes found in cremation ashes. These include small pearl-like spheres called *ringsel*, considered to be signs of the cremated person’s spiritual realization.^35^ Or cases where the cremated practitioner leaves behind their eyes, tongue, and heart, symbolizing a triad of Buddhist qualities that a great practitioner or teacher embodies. I’ve come across cases of this in my fieldwork, where the body was burnt for days until only ashes remained. In one case I examined, the heat was so intense that the brass vajra (representing a diamond or thunderbolt scepter) and bell ritual implements placed in the body’s hands melted, yet the practitioner’s eyes and tongue apparently were not burnt. Other extraordinary postmortem displays include images of Tibetan Buddhist mantras and deities imprinted onto the charred skulls and bones left behind in cremations.^36^ There are also environmental displays associated with saintly deaths such as clear skies, certain kinds of cloud formations or light phenomena.^37^

An epistemological approach seems insufficient to account for such phenomena, where moral, religious categories and symbolism seem to manifest in physical, biological reality, *even postmortem*. If these phenomena indeed occur—as the tradition and many modern-day observers claim—we are not looking at mere social construction, discourses, interpretations or representations regarding an underlying reality most accurately described in scientific terms. Rather, we are faced with a situation where the very fabric of reality seems to manifest in accordance with these Indo-Tibetan forms—which is not to say it could not manifest in accordance with other cultural forms—thus calling for an ontological approach. Again, this entails collapsing epistemology and ontology, so that different ways of conceiving-perceiving contribute toward producing different ontologies, a point that I’ll further elaborate on below.

Such a radical conclusion resonates with ideas proposed by Holbraad and co-authors Amiria Henare and Sari Wastell in their introduction to *Thinking Through Things* (2007). The core proposal of their introduction is to collapse the distinction between meaning or concepts, and things or matter. This follows from seeing through and rejecting a Eurocentric, modern naturalist ontology, again, of a nature of “things” out there, and their different epistemological, cultural representations. What is argued for is the inseparability of things and concepts. A famous example from Holbraad’s own contribution to the collection of essays illustrates this: a Cuban diviner’s powder *is* power rather than just representing it, as traditional anthropology with its attendant modern ontology might have it. According to the traditional approach, “they” just *believe* the powder is power, while “we” *know* that what is really there is this material substance, powder, to which they attribute different meanings, such as power. This will not do, for Holbraad. Like Viveiros de Castro, he wants the others’ concepts to dictate the terms of engagement, and what kinds of things/concepts emerge from ethnographic encounters. This raises the tantalizing notion that reality itself—or at least how we experience it—is in some sense conceptual, but the authors do not elaborate or make any kind of sustained argument for this.^38^

## Buddhist Worlding

Buddhist philosophy, however, has a lot to say about concepts and their relation to reality. The Indian Yogācāra philosophical system and its later epistemological (pramāṇic) developments in the thought of Dharmakīrti emerge as particularly productive traditions to engage in relation to our concerns.

My engagement with Buddhist philosophy is carried out in the spirit of anthropologists like Viveiros de Castro, who creatively borrow, stretch and rework “indigenous” concepts for their own philosophical, anthropological purposes.^39^ While I hope to convey ideas of certain Buddhist philosophical strands, I too am not aiming for precise, literal presentations, but creatively deploy different interpretations and strata of thought as befits my argument.^40^ The ordering of Buddhist ideas, then, does not strictly follow their historical development. In broad brushstrokes, I move from epistemological presentations of Dharmakīrti to idealistic levels of analysis that align with earlier Indian Yogācāra views, and finally come to much later Tibetan tantric appropriations of Dharmakīrti and Yogācāra that land us in a groundless ultimate beyond ontologies, allowing for multiple onto-epistemologies. Although I get into more explicitly Tibetan terrain toward the end of this section of the article, my treatment of all this material is influenced by Tibetan interpretations, and especially those of the Seventh Karmapa Chödrak Gyatso*.* While his interpretations are far from orthodox and certainly not agreed upon by all schools of Tibetan Buddhism, I take his views to somewhat align with my ontological arguments.^41^ Moreover, taking a Tibetan lens on Indian philosophical material is not inappropriate considering the Tibetan Buddhist focal point of this article (and collection). Indeed, Chödrak Gyatso’s tantrically inflected readings also directly relate to the state of *tukdam,* as we will see.

In what amounts to the most comprehensive Indian Buddhist philosophical system, Yogācāra, and its later pramāṇic developments,^42^ phenomenal reality emerges as conceptual. Subjects, objects, and entire worlds emerge based on an illusory subject-object dualism,^43^ which lays the basis for variegated phenomena, carved out or delineated in dependence on deep-seated habitual patterns of conceiving-perceiving. 

According to Dharmakīrti,

The arrangement of things as different is based on that distinction [between subject and object]. When that is a distortion, their difference is also a distortion. Moreover, there is no defining characteristic [of things] outside of the phenomenal forms of subject and object. Since they are empty of a defining characteristic, they are shown to be essenceless.^44^

Distinct things emerge from a conceptual matrix based on subject-object duality.^45^*Pramāṇa* or the “valid cognition” epistemological tradition associated with 5th and 6th century Indian Yogācāra philosophers Dignāga and Dharmakīrti developed an intricate nominalism showing how entities are constructed through concepts and language—and as such, exist only nominally or conventionally. Dharmakīrti is well known for his arguments on valid cognition on the level of conventional reality, where he asserts causal efficacy as the hallmark of being existent. In this domain, direct perception and inference are the means to valid knowledge and the measure of truth.^46^ In Dharmakīrti’s technical terms, direct sensory perception is non-conceptual and gives us access to the real. However, perception in its commonly understood sense—perceiving spatiotemporally extended entities, or “what is” for us, in our human-experiential world—turns out to be conceptual and thus in a way fabricated.

In this regard, Dharmakīrti’s theories have found purchase in contemporary cognitive science and cross-cultural philosophy of mind debates about the scope of the conceptual and non-conceptual, for instance in acts of perception.^47^ While explicating the complexities of Dharmakīrti’s thought is beyond the scope of the present article, suffice it to say that since perceptual categories are constructed by a process of exclusion (*apoha*) from that which is regarded as other to the percept under the influence of past experience, common acts of perception are conceptual (in a minimal sense). As Evan Thompson notes, in Dharmakīrti’s minimalist view on concepts, perceptual recognition is conceptual cognition, also influenced by motivational and affective factors, as well as past experiences, etc.^48^

Could different ways of conceiving-perceiving then bring about different ontological bodies? Or different cultures different and incommensurable ontological worlds, as some anthropologists seem to be claiming?^49^ At first sight, Dharmakīrti comes across more as an epistemologist than ontologist (according to our anthropological characterizations), arguing that the same causal reality can be apprehended in multiple ways. Much of his writing operates on a level of external realism, where an underlying reality is conceptually chopped-up, as it were, into different entities, depending on beings’ aims, needs, and predispositions. In this way, the same reality can be perceived differently by different beings, for whom things are real or validly apprehended insofar as they carry out their causal function, which again, is relative to the conditioning and aims of the conceiver-perceiver.^50^ (For instance, acupuncture points on meridians are real in Chinese medicine, efficacious as they are in predictably relieving certain symptoms.) Applying Dharmakīrti in this way seems rather compatible with the approach taken by epistemologically inflected medical anthropology (including Tidwell in her contribution to this collection), tracking different efficacies in differently delineated biocultural entities nonetheless based on a common causal reality “out there.”

Yet as is well known, Dharmakīrti’s philosophy operates on multiple levels of analysis or truth. When we move to his final idealist Yogācāra level, external reality is denied. All that appears is mind, or transformations of consciousness. As in George Berkeley’s arguments over a millennium later, matter does not exist independent of being perceived. In the Yogācāra view that Dharmakīrti also ultimately espouses,^51^ the physical world can be reduced to phenomenal appearances, emerging from a dynamic system involving subject-object dualism and different aspects of mind. Arising out of this dynamic, the body and physicality feed back into and condition this complex that creates our experience of the world. This is a Yogācāra version of dependent origination.

According to Yogācāra thinking, our particular human embodiment and sense faculties predispose us to similar forms of experience (even if the form experience takes is not “objective,” but rather the product of our embodied interaction with sensory flux), which would presumably also include experiencing bodies in similar ways. Yet bodies are also the result of deep habitual patterns, including conceptual and linguistic ones, which could open the way for ontologically different cultural bodies. Contemporary Yogācāra scholars such as William Waldron have emphasized the sociocultural elements also at play in the way worlds arise in Yogācāra philosophy, and how the intersubjective world of human language and culture feed into this worlding.^52^ Predispositions^53^ created by images, names, and concepts also influence the forms objects take in our experience; past impressions made by speech and language condition the way manifest forms of consciousness arise as different kinds of phenomena. As Waldron writes:

since language is a social phenomenon, these collective influences constitute the so-called ‘shared aspect’ (*sādhāraṇa*) of our subliminal mental processes (*ālaya-vijñāna*), whose correlative object is, accordingly, an indistinct ‘shared common world’ (*bhajāna-loka*). That is, we humans live in a shared, species-specific world because we have both similar cognitive capacities, such as human sense faculties, as well as similar influences from shared culture and language, which are inextricably social. Thus, Yogācāra theory describes not just the cognitive construction of objects appearing to individuals, as in early Buddhism, but also the unconscious construction of the implicit, shared world of our species (*bhajāna-loka*) (2008, p. 9).

In his work on early Indian Yogācāra approaches to intersubjectivity and metaphor (drawing on philosophers like Vasubandhu and Sthiramati), Roy Tzohar also highlights how concepts and linguistic activity shape physical-experiential worlds.^54^ “It is... the inescapably intersubjective nature of our conceptual activity and language use, seen as causally efficacious, that accounts for the common content of our experiences and of the world that we inhabit” (Tzohar, 2017, p. 349).^55^ In this mentalistic framework, the kinds of forms we are and the worlds we inhabit are mental appearances, which are the end products of a causal mental flow (ibid., p. 347). Here, each type of being is explained as constituting “a particular mental causal continuum, a certain mind-stream *(saṃtāna)* within the general causal mental flow...” (ibid., p. 346). The patterning of a mental causal continuum corresponds to a certain limited range of possible experiences of what *is*, or affordances for action, for any given being. In a classic example, humans (as constituted by certain mental causal patterns) see a river of pure water, while hungry ghosts (whose mental causal patterns differ in some respects from humans’) see the same locus as a stream of pus. What explains similarity and discrepancy of perception—and worlds—is the extent to which beings’ causal mental streams are similar and different (as formed through their past actions, experiences, intersubjective influences including language and culture, etc.^56^). To the extent that we share similar mental causal streams, we perceive similar worlds and forms, of human bodies for instance.^57^ To the extent our causal streams differ, these will be perceived in different aspects or functions—in the case of our example, this might mean not even perceiving bodies at all. This account of intersubjective agreement and disagreement does not only apply to traditional Buddhist realms (of humans, hungry ghosts, animals, and so forth), but also within realms, and therefore between e.g., different humans or human groups.^58^

It might seem like we are still retaining a common underlying “landscape,” even if it is relegated to the sphere of the mental, as some kind of shared mental causal soup out of which distinct continua emerge. Any such ground could however be undermined by Dharmakīrti’s quiet admission that causality itself is just a matter of convention, or that the relationship between cause and effect is merely “made by thought.”^59^ As noted by translator Khenpo David Karma Choephel in *Establishing Validity: The First Chapter of Karmapa Chödrak Gyatso’s Ocean of Literature on Logic and the Corresponding Chapter from Dharmakīrti’s Commentary on Validity*, “the statements frequently encountered in the secondary literature that Dharmakīrti considers only the causally efficient to exist ultimately should be taken with a grain of salt, for a close examination shows that Dharmakīrti only makes such a statement on a lower, provisional level of analysis” (note 29 to the Translator’s Introduction, p. 299).

What remains in Dharmakīrti’s final Yogācāra level of analysis is revealed when subject-object duality and all attendant conceptual fabrication is cut through: non-dual, non-conceptual reflexive awareness (*svasamvedana*), which is the very basic nature of mind and reality. Somewhat counterintuitively, here the ground recedes furthermore, as this ultimate transcends space, time, unity, and difference, and thereby causality as well as phenomenal worlds and their variegated forms.

Edging further into groundlessness, let us look at Dharmakīrti through tantrically inflected Tibetan readings that highlight his decidedly antirealist bent. In contrast to realist interpretations among the Geluk sect that has dominated the Tibetan religious and political landscape to this day, I turn to Kagyü interpretations, and particularly those of Karmapa Chödrak Gyatso (1454–1506)^60^ who interprets Yogācāra and Dharmakīrti in a way that provides a basis for tantric meditations such as Mahāmudrā (also implicated in *tukdam*)*.* According to Chödrak Gyatso, Dharmakīrti’s final intent is that of the Great Middle Way (Mādhyamaka) of the Yogācāra, which also emphasizes the *emptiness* or lack of substantial existence of non-dual, self-reflexive awareness.^61^ In this view, Yogācāra and Dharmakīrti are not to be identified as idealists granting consciousness true existence. Nor is some kind of common (mental) causal reality, or dependent nature in Yogācāra terms, posited as foundational. As Chödrak Gyatso writes, “saying that the dependent is truly established in the Yogācāra tradition is merely the laziness of confusing Idealism for the Yogācāra Middle Way” (Chödrak Gyatso, p. 138).

The truly established, or ultimate, is beyond existence (ontology) and non-existence, mind and matter, utterly ineffable and out of reach of the conceptual mind and its dualisms. It is the union of emptiness and luminosity, or self-cognizing knowing, which is identified as the Great Middle Way. In the contemplative domain, this is equated with Mahāmudrā meditation or “ground luminosity” which the *tukdam* meditator abides in at the time of death. In true tantric fashion, dualistic permutations of consciousness are in fact expressions of this fundamental empty luminosity (in this context also called a “ground”) so that the essence of dependent awarenesses—the conditioned, dynamic causal complex giving rise to experiences—is the empty luminous nature of mind, which is merely distorted, or refracted, into phenomena (or subjects, objects, and worlds).^62^

We have moved beyond idealism and arrived at a groundless ground transcending existence. At this point, we might ask why we should take seriously this Indo-Tibetan Buddhist vision, which Buddhist Studies scholars might argue is just a particular historically located cultural formation. From an ontological anthropological perspective, such a question betrays an epistemological approach giving priority to “a real” understood in academic, Euroamerican, historicist terms. The anthropological question in relation to ground should be: whose ground? According to what, or whose, cultural criteria of truth or knowledge do we operate and ground our claims? When navigating Tibetan Buddhist phenomena, it is anthropologically fitting to draw on Tibetan Buddhist concepts, worlds, or in this case, the ultimate insubstantiality of these. This is not to underplay the potential validity, in more cross-culturally far-reaching terms, of these Tibetan Buddhist views, as arrived upon through philosophical enquiry and contemplative methods. Indeed, the tradition I employ has developed sophisticated reasonings and contemplative practices that lead to the kinds of insights that can here only be described in most cursory, impressionistic terms. To be compelling, these views really require working through such reasonings and contemplative practices, which is obviously not possible here.^63^ As earlier argued—also drawing on medical anthropologists like Mol—certain versions of the *real* (or the body) come into being in dependence on enacting particular kinds of theory and praxis, including attending to the body, mind, and other phenomena in different sensory, affective ways. To really arrive at our groundless ground would then require much more work than this article can perform.

Such fundamental groundlessness beyond existence and non-existence (or both existence and non-existence, or neither) is however relevant to our ontological concerns.^64^ Again, with any common ground, or existence, denied, it makes sense to assert ontologies in the plural, as conceived-perceived by different beings. In other words, to *exist* is to exist for a conceiver-perceiver, according to their needs, aims, habituation, conditioning, etc., which will vary from group to group. Unable to secure any fundamental ontology, the conceptually conditioned worlds that arise interdependently with beings then constitute the only meaningful sense of ontology*,* or rather, ontologies.

With a bit of maneuvering, squeezing, and pulling—in line with the ontological turn penchant for employing “indigenous” ideas for concept creation and experimentation—there could then be a way to think multiple cultural ontologies through Yogācāra and some of its later Tibetan appropriations, although no such idea is put forth, to my knowledge, in Yogācāra texts. Multiple cultural ontologies also seem to fit Dan Lusthaus’ framing of Yogācāra, where all ontologies are epistemological constructions (2014, p. 9). What different worlds, bodies or deaths in their different configuration*s* fundamentally *are* comes down to onto-epistemologies, where epistemology and ontology collapse.

## Ontologies, Indeterminacy, and Complementarity

Ultimately, there is no single fundamental body, landscape or even physicality out there. It seems we could hardly get further from a scientific, physicalist view of the real, or nature. In this Buddhist view, the ultimate nature is certainly not an objective physical or material universe, which itself is seen to arise in dependence on delusional habitual tendencies of conception-perception, also influenced by collective aspects like language and culture. Then again, the XIV Dalai Lama has repeatedly spoken about the similarity between Yogācāra views and quantum physics.^65^ Drawing on decades of dialogue with scientists, he has noted how both systems show the lack of objective reality, and how this depends on observation. Interestingly, this resonates with feminist science studies scholar and theoretical physicist Karen Barad’s work, where she elaborates on quantum physicist Niels Bohr’s ideas to come up with a theory that could account for multiplicity and incommensurability (2003, 2007). Barad draws on Bohr’s ideas of indeterminacy and complementarity, as for example seen in the dual nature of light (as particles or waves). Light should not be thought of as a stand-alone entity in the world “out there;” rather, it is inextricably bound up with what Barad calls “agencies of observation,” or the specific material practices used to measure it. She notes, “As a matter of principle, there is no unambiguous way to distinguish between the ‘object’ and the ‘agencies of observation’” (2007, p. 114). The world, then, is not composed of independent objects, but what Bohr called “phenomena.” These signify the wholeness of interaction between the “objects” under investigation and the “agencies of observation.” Phenomena, rather than objects, are the basic units of existence, “ontologically primitive relations… without pre-existing relata” (333). Resonances with Yogācāra thought are obvious, and abundant.

The object-in-itself, for example light, is “indeterminate,” until enacted through this or that experimental apparatus as either particle or wave. In Bohr’s terms, the wave and particle properties of light are “complementary,” or mutually exclusive. Barad applies Bohr’s observations to reality at large, in what she calls her “agential realist ontology.” Here, different apparatuses of observation and practice bring forth different phenomena, which are constitutive of reality. What we perceive as bodies or matter is also brought forth through such dynamics or “intra-activity,” as is spacetime itself.

In anthropology, Paul Nadasdy has used Barad to come to grips with “the multiple worlds [ontologies] thesis” which he finds intolerable, as do colleagues like David Graeber (Nadasdy, 2021; Graeber, 2015). Nadasdy sees his task as “the laudable political project of decolonizing knowledge” (that is, taking seriously different cultural claims about the world without subjugating them to Euroamerican standards or ontologies) from “the morass of multiple worlds”^66^ or, quoting Graeber, the “ontological anarchy” that the proliferation of different cultural worlds would lead to. Nadasdy starts by evoking Mol’s work and, guided by Barad, translates Mol’s argument on multiplicity into Bohrian concepts of indeterminacy and complementarity. Is atherosclerosis pain when walking? Or blocked arteries seen through a microscope? “Only once a particular set of material practices has been chosen can atherosclerosis be enacted as one or the other. In Bohr’s terms, these mutually exclusive versions of atherosclerosis-in-practice are complementary.”^67^ Indeterminacy, Nadasdy maintains, does not require multiple ontologies, only incompatible material and semiotic practices. “Different practices do not enact different worlds… but complementary phenomena in the Bohrian sense (which are relational processes, not things), each of which gives us access to an aspect of “reality” that the other forecloses.”^68^ Nadasdy admits that different cultural worlds may traffic in fundamentally different entities but hopes to salvage one “reality” through indeterminacy. Different cultural enactments then reveal different aspects of this reality, even if no single “objective” nature can be reached.

It is debatable whether Nadasdy or Barad’s approach gives us more ontological ground to stand on as compared to multiple cultural ontologies. Both approaches imply an incommensurability, or complementarity if you will, of entities depending on different practices, modes of observation, or conceiving-perceiving. This raises important questions about possibilities of translation between very different knowledge systems on the body and death—as we have with Tibetan Buddhism and the scientific Tukdam Study, with its attempts to translate Buddhist ideas and observations into scientific terms. Translation requires common ground.^69^ Yet could it be, as implied in foregoing discussion, that these systems are not in fact engaging the same terrain, but rather, each is engaged in co-producing its own particular landscape (perhaps with some correspondences or cross-over)? In the online conference on death in Tibetan traditions organized by Yale University,^70^ Thubten Jinpa was excited by developments in the Tukdam Study. Originally trained as a Geshe^71^ in the Geluk tradition of Tibetan Buddhism, Jinpa is known as the Dalai Lama’s principle English translator and major figure in the science-Buddhism dialogues. Nonetheless, Jinpa questioned whether it was possible to get a “synthetic description” of the two ontologies at play in the study. While insisting that there could only be one thing happening (in death or *tukdam*)^72^, he wondered whether the two ontologies could actually ever come into contact. With decades of experience at the frontlines of this cross-cultural encounter, Jinpa’s remarks could be taken to express a kind of weary frustration with efforts to make the two systems coincide, though they harbor such different epistemologies and ontologies of body, mind, life, and death.

## Regimes of Truth and (Media) Invisibility of Tibetan Deaths

In the Buddhism-science dialogues, science has not been very receptive to Tibetan ideas diverging too radically from a materialist, and brain-centered scientific paradigm.^73^ Tibetan Buddhist ideas have had to be expressible or operationalizable into scientific idioms to gain acceptance, and crucially, validation among the modern educated elite for whom scientific knowledge tends to rule supreme. While the Tibetan side has also been reluctant to revise its deeply held views—say, about mind as immaterial and ultimately transcending the brain-body complex—the power asymmetry between modern science and Tibetan knowledge is clear enough. The spectacular success of the natural sciences has certainly contributed to the cultural power science commands today. While it does not necessarily follow that something is more real just because “it works” (according to particular criteria set by a given group), many are easily persuaded to accord a more fundamental reality to a scientific world (or biomedical body) which then easily petrifies into *the* real. Certainly such ontological fixation is common outside these kinds of academic discussions, as I found in the course of making my documentary.

To end where I began, let me then briefly return to *Tukdam: Between Worlds,* or its editing process, to be more specific. Reactions to a scene that did not make the final cut of the film are illustrative of this power asymmetry as well as fixation on a single (scientifically framed) ontology. In this removed scene, we saw Tukdam Study Manager Dylan Lott in his role as anthropologist querying Tibetan medicine doctor Ngawang Jinpa about subtle winds (dynamic psychophysical forces) implicated in aspects of life, and thus death, in the Tibetan medical system. As Dylan, Dr. Jinpa and assisting Tukdam Study team member Tenzin Desel await an old monk who has volunteered as a control subject for physiological measures, their discussion unfolds thus: 

*Dr. Jinpa (Desel translating for Dr. Jinpa):*

This heat energy which keeps us alive, which maintains this life form, is called *sok* (*srog*). So this *sok* is just for a lifetime. It ends as soon as we die, and for the next life it’s a different *sok*. But life-sustaining *lung* [wind] is… coming from the previous life, and continues to the next life. This is the big difference.

*Dylan:*

So the warmth and heat that comes from the *sok* is not the same kind of warmth and heat we feel around the heart-center for someone who is in *tukdam*?

*Dr. Jinpa:*

No, it’s not the same. Life-sustaining *lung*… the subtle life-sustaining *lung* resides within the heart.

*Dylan:*

The physical heart, or the…?

*Dr. Jinpa:*

Yes, the physical heart.

This scene elicited a strong negative reaction from a commissioner of the documentary,^74^ who had been somewhat dismissive of Tibetan claims throughout the filmmaking process. Up to this point, separate scenes where Tibetans give their take on things, juxtaposed with scenes of the scientists giving theirs, had been passable. However, the commissioner appeared to find the above scene unpalatable, as it seemed to show a Western scientist assenting to Tibetan views. From what I gathered, it was problematic to present such “unscientific” claims as something to be seriously entertained, considering the commissioner’s remit to educate the public.^75^

Bearing in mind earlier discussion, could we surmise that the commissioner could just about accept separate presentations of what were intuited to be incommensurable ontologies (in separate scenes or interviews), but to bring them together into the same scene—and person of Dylan—was just too much to hold simultaneously?^76^ Despite my intention to present balanced takes on the Tibetan and scientific knowledge systems and ontologies in relation to death and *tukdam*, and the fact that after more than a decade of research the Tukdam Study had achieved little in terms of scientific results or explanations,^77^ it seemed the commissioner could not see that the Tibetan Buddhist explanation—or ontology—might hold some validity in its own terms, independent of scientific validation. This media gatekeeper may have concluded that the scientific protocol probably just needed to be developed so that *tukdam* would eventually be explained in terms of “Western,” “empirical” science. From a position of ontological incommensurability, this may very well be the case: if probed deeply enough through scientific means, a scientific explanation may one day emerge. Whether it has much in common with the Tibetan conception-perception is another matter—as is whether this should take precedence over the Tibetan Buddhist version of *tukdam.*

We see how invisible these Tibetan bodies and deaths can be to a Western medical—and media—gaze, which, as Kuriyama, Farquhar and others have highlighted, privileges the spatial, visual, and increasingly, quantitative, technological measurement. Finnish indigenous studies scholar Pirjo Kristiina Virtanen and medical anthropologist Marja-Liisa Honkasalo further trace out such tendencies in their paper on evidence work^78^ in relation to entities invisible to modern science and its attendant Euroamerican onto-epistemology (Virtanen & Honkasalo, 2020). This onto-epistemology dominates at the level of state authorities globally where it is practiced in a strikingly uniform manner, leading to a power asymmetry between such authoritative forms of knowledge and other kinds of onto-epistemologies. Drawing on Christine Taylor (2007), Virtanen and Honkasalo note how the material and visible world (interpreted according to certain frameworks and methods) have come to constitute the grounds for the criteria of truth. The power of this regime of truth, and its *real,* was felt by many of my Tibetan interviewees and collaborators in the Tukdam Study. For instance, senior Men-Tsee-Khang (Tibetan Medical Institute) doctor Tsewang Tamdin complained to me about Western science’s narrow fixation on the visible. Just because something is invisible does not make it unreal, he remarked.^79^

The lifelike physical appearance of a *tukdam* body is quite obvious to everyone^80^ —which makes it so fascinating in this context and apt for film treatment—*unlike* the Indo-Tibetan tantric body, mind, and many of the related theories and practices within which *tukdam* is produced. It is difficult to make visible such bodies, deaths, and their associated knowledge systems in a regime of truth or evidence where certain forms of visibility, quantification, and measurability set the grounds for valid knowledge. Some of the documentary commissioners continued to see the film as a story of religious belief versus empirical scientific knowledge, despite my insistence that it is about the meeting of two different knowledge systems with widely differing background assumptions, epistemologies, and ontologies. Even though the film, as I see it, presents them with the Tibetan body, death process, and knowledge system in some detail, many insist on viewing it through a dichotomy of religion and science arising from a particular European historical context.^81^ Anything not conforming to biomedical standards of evidence is easily relegated to “belief,” “religion,” or worse, “superstition”—or rendered invisible. We see the same body, but we see different things. Or we see the same film with the same body, and we see different things.

The regime of truth and attendant ontology which reigns at the academy and at levels of state authorities globally is also reinforced by broadcasters and the mainstream documentary film world. In Yogācāra terms, we might say that this lays down and strengthens shared, collective imprints that contribute toward shaping a certain experience, and thus ontology of the world. Such are the challenges I face as a documentary filmmaker and public intellectual, trying to bring visibility to different cultural worlds, bodies, and death processes.^82^ I hope this work can help loosen, if ever so slightly, a fixation on a singular (in this case scientific) real.

I would like to conclude with a related point on the general pertinence of an ontological approach—such as I have tried to outline here—that extends beyond the field of *tukdam* research, or the complex historical-political factors influencing formations of ontologies and their power relations. Here, I will again draw on Buddhism, or rather return to its grand vista of groundlessness. From a Mahāyāna Buddhist philosophical perspective (which Tibetan Buddhists also adhere to), holding onto any real is a distorted view driven by habitual attachments and fixations that lead to undesirable soteriological consequences (as well as the arising of particular worlds, bodies and deaths). Importing and transforming this principle into ontological and medical anthropological registers, I suggest that ontological attachments are liable to make us less open, and sensitive to different worldings and what these might have to offer. This is another reason to question the idea of one landscape and different epistemological ways to access and represent this. If one is fixed on the view of one reality out there, it may be more difficult to see this as multiple and give other cultural (for lack of a better term) worlds their due. A fundamental groundlessness or *śūnyatā—*the state in which a *tukdam* meditator abides—allows for the arisal of many grounds.

## Footnotes

1 As documented by the “Tukdam Study” led from the Center for Healthy Minds at the University of Wisconsin-Madison and reported by more than a dozen medical doctors I have interviewed during my field work in India, Nepal, the U.S., and France since 2014. By and large, these medical doctors also confirmed many of the physical signs of *tukdam* and asserted that the cases they examined defied their expectations of what occurs to a body after death and were generally unlike anything they had seen before.

2 Aspects of *tukdam* will emerge in course of this article, but I will not be giving a systematic detailed presentation of what this is from Tibetan Buddhist points of views, which are indeed multiple. Related esoteric practices surrounding death and dying that developed and are still practiced in Tibetan communities may be roughly divided into two predominant groups: those that grew out of the Indian tantric tradition of the *Guhyasamāja-tantra*, and those that developed in Tibet proper under the umbrella of the contemplative tradition known as the Great Perfection (*Dzogchen*). (I’m grateful to Jacob Dalton for this formulation). The phenomenon, and some of the theories and practices related to it are described in more detail in this Special Collection’s other contributions, which also illustrate in ethnographic detail ways in which *tukdam* is enacted in contemporary Tibetan (exile) communities, as well as its figurations and contestations around the scientific Study. As far as I know, the first and to date most extensive academic study on *tukdam* (in a language other than Tibetan) is my 2017 master’s thesis *Resting Between Worlds: The Ontological Blurrings of Tukdam*. Co-contributor Tenzin Namdul’s (2019) doctoral dissertation on ideal Tibetan deaths also looks at *tukdam*. In 2021, *Frontiers of Psychology* published the first scientific paper summarizing the findings (or rather lack thereof) in the UW-Madison lead Tukdam Study to date (Lott et al., 2021).

3 The film depicts the Study up to 2020, before some of its collaborative developments between Western and Tibetan contingents as described by Tidwell in her contribution to this collection. The Study now also involves Russian and Indian scientific partners.

4 Titled *Tukdam Phenomenon: Paradigms in Shaping Ontological Realities, Discourses with Science, and Tibetan Cultural Identity*

5 *Precarity of Death: Exploring the Boundary between Death and Life in Tibetan Traditions,* Dec 3, 2021.

6 In a way, my documentary *Tukdam: Between Worlds* could be considered as an ethnographic complement to this theoretical text. (The film is available e.g., on Amazon, Apple TV, Google Play, and Vimeo.) More ethnographically oriented texts drawing on my film- and fieldwork related to *tukdam* are forthcoming.

7 This is the title of the 91-minute feature version; a shorter television version with different titles, depending on territory, was broadcast on several European channels in 2023 and 2024. The documentary is a relatively big international co-production with several European broadcasters, national film foundations, and other film funders involved.

8 One traditional sign of *tukdam* is warmth at the heart area—where the subtle energetic aspect of the subtlest mind is said to reside after death—reported to persist for days, or as long as the deceased stays in *tukdam.* This is not only reported by Tibetan observers. Several medical doctors interviewed for my documentary and fieldwork reported feeling this warmth. These included doctors who looked after the 16th Karmapa, one of the most important Tibetan spiritual leaders of the 20th century. He died in a hospital in Zion, Illinois in late 1981. Dr. Raj Kotwal and Dr. Mitchell Levy (who since became Rhode Island Hospital Intensive Care Unit director and Professor of Medicine at Brown University) both reported feeling warmth around the Karmapa’s heart area for several days after he was declared dead. To quote Levy, “The area around the heart, when you felt it, put your hand on his chest, still felt warm, for the first two to three days after he died. Which obviously from a Western point of view is not consistent with what we would expect” (interview in Providence, May 2014).

9 Perlman therefore speaks from his experience of earlier phases in the Study, which he managed up to 2013.

10 See, for example, Adams (2001, 2005, 2006).

11 Adams, unpublished paper *Using Medical Anthropology to Think About the Local Body in Tibetan Medicine* (2015).

12 Referencing medical anthropological scholarship, Eduardo Kohn has noted how “something about the body forces anthropologists onto ontological terrain when they turn their attention to medicine” (2015, p. 315).

13 See, for example, Kohn (2015) and Holbraad and Pedersen (2017, pp. 30–68).

14 Descola (2013).

15 While much ontological anthropological scholarship—including its criticism of epistemology—derives from critiques of modern Western nature-culture ontology that can be associated with scientific realism, it is worth pointing out that not all epistemological approaches need subscribe to a scientific realism. Co-contributor Tawni Tidwell, for instance, places equal emphasis on a scientific “map” of reality and an Indo-Tibetan one drawing on Tibetan medicine and interpretations of Buddhist *pramāṇa* or valid cognition theory. While the “landscape” need not then be a scientific one, asserting a landscape and its different cultural representations bears the hallmarks of an epistemological approach as critiqued by some ontological anthropologists. Moreover, although the assumptions of scientific realism and an epistemological approach can be set apart, these tend to converge and dominate in contemporary academia, the media, and among state authorities as I discuss toward the end of this article.

16 Holbraad and Pedersen (2017, p. 6).

17 To take this concept out of context; Viveiros de Castro uses it to describe a certain kind of Amerindian animist ontology (e.g., 2014). Such borrowing of concepts and creative concept formation is very much part of Viveiros de Castro’s project, aiming to liberate anthropology and philosophy from their Euroamerican constraints.

18 This is an all too brief presentation of a vast subject. For more elaboration, see e.g., Jeffrey Hopkins’ *Emptiness Yoga: The Tibetan Middle Way* (1987), which lays out a Geluk systemization of dependent origination (which this school equates with emptiness).

19 It is quite remarkable that such resonances seem to have gone unnoticed.

20 Such an interpretation and emphasis of dependent designation as a form of dependent origination has been especially emphasized by Je Tsongkhapa and the Geluk school of Tibetan Buddhism (e.g., Hopkins, 1987).

21 E.g., in Bates (1995, pp. 205–234).

22 Dreyfus and Taylor (2015).

23 Farquhar (1991, pp. 385–386).

24 (ibid., p. 386).

25 (ibid., p. 372). Kuriyama’s *Expressiveness of the Body* offers a historical analysis of how the West ended up with its anatomical fixation on discrete, spatial entities—which arguably yielded anatomy, as we know it.

26 I am using the term “Western” here as this has been used in opposition to Traditional Chinese Medicine in China (i.e., 中医—Traditional Chinese Medicine; 西医—Western Medicine).

27 Kaufman (2005, p. 62).

28 (ibid., p. 293).

29 In this context, *tukdam* could be posited as a liminal state of “life-in-death,” also confounding and blurring our definitions of death and highlighting their cultural, conventional bases.

30 “A definition of Irreversible Coma: Report of the Ad Hoc Committee of the Harvard Medical School to Examine the Definition of Brain Death.” *JAMA* (Beecher et al., 1968).

31 In *Twice Dead,* Lock shows how in Japan there has been much more debate than in the West about definitions of brain death and personhood, as well as the commodification of bodies, and concern over possible influence of organ transplant demand on declaring death. And, as Lock points out, this is in a highly developed, modern, and well-educated society.

32 Both in “objective” and “subjective” domains. In the latter, we could mention the example of coming to recognize and rest in “the nature of mind”—a non-conceptual, insubstantial state of cognitive lucidity or pure knowing—said to be also revealed at death; or practicing gathering the psychophysical “winds” into the central channel, also said to occur at death and precipitating an experience of the nature of mind.

33 See e.g., Daston and Garrison (2010) and Saphin and Schaffer (1996).

34 Viveiros de Castro (2003, p. 19).

35 Tidwell and Namdul describe instances of *ringsel* found after cremations in their contributions to this collection.

36 Also described by Tidwell in her contribution to this collection.

37 For a history of such postmortem displays including relics in Tibetan Buddhism, and particularly its *Dzogchen* traditions, see David Germano’s contribution (chapter 3) in *Embodying Dharma: Buddhist Relic Veneration in Asia,* a collection of writings Germano edited with Kevin Trainor.

38 In response to criticism to the manifesto that was the introduction to *Thinking Through Things*, Holbraad, with Morten Pedersen, toned down some of the more controversial claims, for instance, relating to radical difference and multiple ontologies/worlds. *The Ontological Turn: An Anthropological Exposition* (2017) then gives an updated version of the ontological turn, where ontology is presented more in terms of a methodology. The emphasis is on using ethnographic encounters for concept creation, conceptual experimentation, or for conjuring up possibilities of what could be:"…insofar as this way of doing anthropology is concerned with matters of metaphysics or ‘ontology’ in the philosophical sense of the word, it is so in the service of allowing the object of ethnographic analysis to have a transformative effect on the ontological assumptions the anthropologist brings to it, and in that way contribute to setting the terms for the anthropologist’s conceptualization and analysis of it"(68).

39 In much of his work, Viveiros de Castro does not pretend to be presenting animist Amazonian ontologies in their ethnographically and historically located forms, but is rather upfront about engaging in “speculative metaphysics” (for instance putting Amazonian-derived ideas in dialogue with Deleuze in *Cannibal Metaphysics*).

40 Emerging from highly sophisticated, centuries-long literate and contemplative traditions, the Buddhist thought we touch upon has in any case been interpreted and reinterpreted in multiple, sometimes conflicting ways—as shown, for example, in George Dreyfus’ *Recognizing Reality* that looks at varying Tibetan interpretations of Dharmakīrti’s thought.

41 As the Karmapa, Chödrak Gyatso was the patriarch of the Karma Kagyü tradition of Tibetan Buddhism. In the context of this Special Collection, highlighting Kagyü views also serves to balance Geluk (and to a lesser extent, Nyingma) sources that predominate in the other contributions, thereby conveying a bit more of the variety, and contentiousness, of philosophical views within Tibetan Buddhism.

42 In Tibetan traditions, Yogācāra is often presented as a kind of strawman idealism, and “lower school” compared to the (Prāsaṅgika) Mādhyamaka (or Middle Way). However, there are many modern scholars who consider Yogācāra as the most sophisticated and full philosophical articulation of Buddhist philosophy, and emptiness (e.g., Nagao 1991). In January–February 2022 the head of the Karma Kagyü school of Tibetan Buddhism, the 17th Karmapa Ogyen Trinley Dorje gave winter teachings on Yogācāra or “Mind Only” as it is commonly known in Tibetan Buddhism, also defending its depth, breadth, and profundity rivaling that of Mādhyamaka. Indeed, in Kagyü formulations Yogācāra is often understood as continuous with Mādhyamaka, and the final intent of Yogācāra philosophers is that of the “Great Middle Way” or Mādhyamaka. This view heavily informs the highest meditation system in the Kagyü school, The Great Seal or Mahāmudrā (*phyag rgya chen po*), which is frequently associated with *tukdam* deaths. Arguably the most comprehensive textual source available on *tukdam* is Karma Chakme Rinpoche’s (1613–1678) *Mountain Dharma, Oral Instructions for Mountain Retreat (ri chos)*, and specifically volume four in a recent English translation edition of this comprehensive practice manual of the Kagyü lineage (Khenpo Kartar 2010). Here different kinds of *tukdam* are correlated with twelve levels of Mahāmudrā realization. It could also be noted that tantra and subtle body theory and techniques—involving ontologically distinct Indo-Tibetan mind-body systems comprised of channels, winds and drops or essences—fit more comfortably with a Yogācāra view than Mādhyamaka. For a classic presentation of the tantric Buddhist subtle body (or vajra body, as it is called in this text) and the mind-body relationship, see the 3rd Karmapa Rangjung Dorje’s *Profound Inner Principles* (2014).

43 In this connection, it is interesting to note that Descola (2013) proposes interiority and exteriority (or physicality) and modes of identification based upon these as giving rise to distinctly different ontologies among human cultures.

44 *Pramāṇavārttika* 3.214–3.215, from Catherine Prueitt’s chapter *Is There an Ideal Scientific Image? Sellars and Dharmakīrti on Levels of Reality,* in Garlfield (ed.) (2019, p. 56). For an alternative translation, see e.g., Dunne 2004, pp. 408–409.

45 While my account here focuses on epistemological aspects of Yogācāra thought, it should be noted that Yogācāra was chiefly concerned with a psychological explanation of the mind and its workings (which includes its relation to phenomenal reality). Here, the depth-psychological “ground” consciousness or *ālaya-vijñāna* emerges as a key-concept, explained as a subliminal “store” for impressions, habitual tendencies, etc., that give rise to experiences of ourselves, our bodies and the world. The role of the *ālaya-vijñāna* in “worlding” is especially emphasized in early Yogācāra thought that connects to the Sautrāntika school.

46 In *Pramāṇavārttika* (*Explanation of Valid Cognition*)*,* Dharmakīrti is mostly concerned with elucidating such conventional valid cognition that assumes external reality.

47 Thompson (2020).

48 ibid.

49 E.g., Blaser (2012, 2013) and De la Cadena (2010, 2015).

50 There are resonances here with William James’ “pragmatics of effects” [as termed by Espírito Santo and Blanes in *The Social Life of Spirits* (2014, p. 4)], or more recently, Bruno Latour, for whom things exist in the sense that they produce effects or affect the world around them (e.g., Latour, 2005).

51 Historically Dharmakīrti came later than some of the classic or “early” Indian Yogācāra thought I delve into here. It is important to note that there are significant developments and differences between different strata and thinkers associated with this tradition that are in places getting blurred in my text. Here, I have prioritized certain conceptual points over finer Buddhist distinctions, the sorting of which may not be entirely appropriate in the context of this forum.

52 Waldron (2003, 2008, 2023) and Germano and Waldron, in Nauriyal et al. (2006).

53 In Yogācāra Buddhism, these are also built up over many lifetimes; i.e., they are karmic.

54 Tzohar (2017, 2018).

55 Tzohar also writes, “...linguistic conventions, categories, and conceptual proliferations (*prapañca*)... are responsible for *the shared elements* in our cognitive experiences... it is the shared karmic seeds and impressions mediated by specific conceptual, linguistic, and cultural baggage that account for these life forms’ particular mode of being, for the physical-experiential world that they share, and for the specific content of their experiences” (2017, p. 349).

56 All of which have left impressions on the subliminal storehouse-consciousness or *ālaya-vijñāna,* influencing the forms of consciousness that emerge as phenomenal reality and its variegated forms.

57 or the same spatiotemporal locus—e.g., of a liquid-like stream—in more general terms. In this scheme, we need not preconceive of some kind of shared absolute “space” out there, perceived differently or from different vantage points; rather, everything—including space and time—is experience, a manifestation of mind, in its broadest sense.

58 Sthiramati argues that such a mentalistic Yogācāra account fares better than appeals to external reality in accounting for intersubjectivity. Whereas the Yogācāra account explains both agreement *and* discrepancies in experience/perception, the form of external realism that Sthiramati disputes struggles to explain discrepancies (which can be radical) (Tzohar 2017, 2018). Moreover, this Yogācāra model can explain the simultaneous *overlap* and differences between worlds/ontologies, i.e., how different beings perceive in some respects similar entities—e.g., the overall shape of bodies or a liquidlike spatial locus—which are yet in other respects different, without positing an underlying ontological furniture “out there.”

59 25 d, 26a-c and commentary in the Perception chapter of Karmapa Chödrak Gyatso’s *Ocean of Literature on Logic* on validity (translated by Khenpo David Karma Choephel; publication forthcoming). In terms of commentaries, Chödrak Gyatso draws mainly on Dharmakīrti’s earliest commentators Sakyabuddhi and Devendrabuddhi. The latter’s gloss on cause and effect being made by thought is found in his commentary on the Perception chapter of the *Pramāṇavārttika*, 134b. [Devendrabuddhi. *A Textual Gloss on the Commentary of Validity*. (Pramāṇavārttikapañjikā, Tshad ma rnam ‘grel gyi ‘grel pa). DT Tshad ma che pa, 1a–326b]. See also Dunne (2004, p. 79).

60 The influence of Chödrak Gyatso’s work on Dharmakīrti and *pramāṇa* (the valid cognition epistemological tradition) also extends beyond the Kagyü school. Among important Tibetan thinkers influenced by him are the great Sakya scholar Sakya Chokden and the Nyingma master Mipham Rinpoche. My treatment of Chödrak Gyatso’s material draws largely on *Establishing Validity: The First Chapter of Karmapa Chödrak Gyatso’s Ocean of Literature on Logic and the Corresponding Chapter from Dharmakīrti’s Commentary on Validity* (2016), with translation and notes by Khenpo David Karma Choephel, and to a lesser extent, Khenpo’s as of yet unpublished manuscript on this work’s Perception chapter. I am grateful to Khenpo Choephel—who is also the principal translator of the 17th Karmapa—for our correspondence and his counsel on this material.

61 Among modern scholars, Karl Brunnhölzl recently noted how *svasamvedana* (non-dual reflexive awareness) of the Yogācāra seems practically indistinguishable from the nature of mind of Mahāmudrā, the meditative expression of the Great Middle Way. (Dharma Wisdom Chats on the *Wisdom Publications* website, April 23, 2024: https://wisdomexperience.org/wisdom-dharma-chat-karl-brunnholzl-2023-04-05/).

62 Such formulations can be seen to express innovative Tibetan syntheses of tantra and Mahāyāna Buddhist philosophy, as elaborated for example by 13th–14th century masters 3rd Karmapa Rangjung Dorje and Longchenpa.

63 On the contemplative side, the relevant meditations require specific initiations, empowerments, and long-term, step-by-step practice under the guidance of qualified teachers to reach the views or insights, here briefly described, in deep experiential terms, or “for real” for oneself. Following tradition, the details of such practices, and their insights, should not be shared outside this context, where public forums such as this publication would fall.

64 We could here make a comparison to the way Viveiros de Castro uses his Amazonian-influenced concept of perspectivism to think about fundamental anthropological issues of cultural comparison, knowing the self through the other, etc. Perspectivism has some resonances with the Buddhist perspectival views touched upon here, although it differs in many important respects, emerging from an Amazonian logic of predation (e.g., Viveiros de Castro, 2014).

65 E.g., public teaching in Dharamsala, India, 3 June 2019.

66 Nadasdy (2021, p. 6).

67 (ibid., p. 7).

68 (ibid., p. 9).

69 On issues of cultural translation, see the 2014 issue of *HAU*, Translating Worlds: the epistemological space of translation, edited by Hanks and Severi.

70 Mentioned in the introduction: *Precarity of Death: Exploring the Boundary between Death and Life in Tibetan Traditions,* Dec 3, 2021.

71 Roughly equivalent to doctor of philosophy in Western Academia. “Khenpo"is a similar title in other schools of Tibetan Buddhism, such as the Kagyü.

72 To what extent such an attitude reflects Jinpa’s Geluk realist training in valid knowledge/Buddhist epistemology would be an interesting question to pursue, along with a more general interrogation into the relationship between realist Geluk stances on valid knowledge and their science-positivity (as evidenced for instance in massive science centers opened at the great Geluk monastic colleges in Karnataka, the inclusion of science in monastic curriculums, or the Geluk participation in the scientific Tukdam Study where science-trained monastics are now carrying out data collection in the field). Indeed, I am conducting further research into such issues for future publications. As a point of contrast, I might mention my November 2024 interview with Shedra (monastic college) teacher Khenpo Phurba Wangchuk at the major Karma Kagyü monastic center of Palpung Sherabling in Northern India, where a view quite different to Jinpa’s emerged. In Khenpo’s presentation of *pramāṇa* as well as Mādhyamaka, there is in fact no common object out there for different perceivers seemingly perceiving the same object—say, an iPhone. This would extend to the study of *tukdam*: strictly speaking, there is no common object for the scientists and Tibetan Buddhists, which could in part explain difficulties of commensuration.

73 As illustrated, for example, in neuroscientist David Presti’s (2018) Columbia University Press publication *Mind Beyond Brain: Buddhism, Science and the Paranormal* or Matthew King’s recent contribution to the volume *Buddhism and Healing in the Modern World* (2024) titled *If You Meet the Medical Buddha on the Road, Kill Him! Lobsang Gyatso and the Shadow of the Mind & Life Dialogues.*

74 The documentary had many commissioners/funders from many different countries. The text is rendered in a way that preserves anonymity, which also necessitates writing in rather vague, unspecific terms.

75 In pitching this film (and previous films delving into non-Western worlds) at international documentary film forums, I have found similar attitudes to be quite pervasive. This domain prides itself as promoting liberal values, tolerance and multiculturalism, yet what does not fit the rather narrow liberal bill and mainstream scientific worldview usually does not get funded or widely distributed. Such worlds are rendered invisible at the “top tier” of the documentary industry, which arguably has some influence on views of international publics.

76 It should be noted that the commissioner did not force the removal of this scene, which was taken out for a number of editorial reasons.

77 It has not found EEG activity in *tukdam* meditators (that could explain their lack of decomposition) so far, which the film also tells toward its end (Lott et al., 2021). However, contributions to the forensic field as explored in more recent Study developments appear potentially noteworthy (Tidwell et al., 2024).

78 Adopting the concept from Garfinkel (1967) and Holbraad (2008, 2012).

79 It is important to note that Tibetans are of course also dealing with a visible and material world (in addition to invisible energy channels, states of consciousness, moral influences including karmic forces, etc.). Yet different physical phenomena may here signify, or *be* different things. For example, the lack of decomposition signifies the presence of consciousness—or as in the above exchange cut from the film, different kinds of *lung* (winds)—which is not at all obvious to biophysical science, or for some other Buddhist traditions for that matter. The medieval Chinese Chan tradition, for example, also records miraculously preserved meditators’ bodies. These did not however signify present consciousness, which according to widespread Buddhist doctrine, departs immediately at death to be reborn or to enter nirvana. Here, the body is preserved due to purification by religious practice and virtue accumulated while alive (Sharf 1992, 1999).

80 It is striking that something unusual seems to be going on in *tukdam* for most observers, irrespective of their cultural frame or system of knowledge. Doctors of the 16th Karmapa mentioned in footnote 8, for instance, perceived heat at the heart despite being outsiders to the Tibetan world and its knowledge systems. This might seem to problematize the argument on different cultural ontologies and imply commensuration. The ontological anthropological rejoinder to this might be that what that heat (or nondecay, etc.) *is* is different depending on the cultural world one inhabits—as fundamentally, ontologically different as the Cuban diviner’s power is from powder (to reference Holbraad’s example glossed earlier in this article). Other similar examples abound in literature on ontological politics or conflicts. Mario Blaser, for instance discusses the case of an orca whale who is Tsux’iit or the abode of the spirit of the recently deceased chief of the Mowachat/Muchalaht First Nations people, a being ontologically distinct to the “animal” of Canadian scientists, bureaucrats, or environmentalists (2013); or Marisol de la Cadena’s work on a contested mountain in Peru, who is a powerful sentient *tirakuna* (earth being) for local indigenous people, but a mindless collection of rocks and minerals for government officials, scientists, and leftist activists (2010, 2015). The reason why one party sees a sentient earth being while others see lifeless geology is that they inhabit different worlds, according to de la Cadena. How then is communication, or politics, possible across these seemingly incommensurable worlds? For de la Cadena, the answer is found in Viveiros de Castro’s notion of equivocation: “As mode of communication, equivocations emerge when different perspectival positions—views from different worlds, rather than perspectives about the same world—use homonymical terms [the same word] to refer to things that are not the same” (2010, pp. 350–351). Such equivocation is possible because the different worlds are not totally separate. Here de la Cadena draws on Strathern’s idea of partial connections as well as Mol’s analysis of how multiplicities “hang together.” In the above “Buddhist Worlding” section, I describe a Yogācāra solution for the simultaneous overlap and differences between worlds/ontologies, i.e., how different beings perceive in some respects similar entities, which are yet in other respects different, without positing an underlying ontological furniture “out there.” What explains similarity and discrepancy is the extent to which beings’ causal mental streams are similar and different, as formed through their past actions, experiences, intersubjective influences including language and culture, etc.

81 As traced out, for example, by Stanley Tambiah in *Magic, Science, Religion, and the Scope of Rationality* (1990).

82 In the unlikely case that the commissioners/funders of the film read this article, I hope they are forgiving of my critique, which is not aimed at specific individuals or companies but rather intends to communicate something about the media and documentary film worlds in general, and how they reflect and reinforce prevailing Euroamerican epistemology and ontology (which is to be expected). Considering this media environment, I would like to acknowledge the courage of our commissioners and funders and thank them for supporting such a difficult project, and in this way, also helping to make these bodies and deaths visible (to those who can see them).
